# The Anal Fistula Plug versus the mucosal advancement flap for the treatment of Anorectal Fistula (PLUG trial)

**DOI:** 10.1186/1471-2482-8-11

**Published:** 2008-06-23

**Authors:** Paul J van Koperen, Willem A Bemelman, Patrick MM Bossuyt, Michael F Gerhards, Quirijn AJ Eijsbouts, Willem F van Tets, Lucas WM Janssen, F Robert Dijkstra, Annette D van Dalsen, J Frederik M Slors

**Affiliations:** 1 Department of Surgery, Academic Medical Centre, Amsterdam, The Netherlands; 2 Department of Clinical Epidemiology and Bio-statistics, Academic Medical Centre, Amsterdam, The Netherlands; 3 Department of Surgery, Onze Lieve Vrouwe Gasthuis, Amsterdam, The Netherlands; 4 Department of Surgery, Spaarne Hospital, Heemstede, The Netherlands; 5 Department of Surgery, Sint Lucas Andreas Hospital Amsterdam, The Netherlands; 6 Department of Surgery, Zuwe Hofpoort Hospital, Woerden, The Netherlands; 7 Department of Surgery, Isala Clinics, Zwolle, The Netherlands

## Abstract

**Background:**

Low transsphincteric fistulas less than 1/3 of the sphincter complex are easy to treat by fistulotomy with a high success rate. High transsphincteric fistulas remain a surgical challenge. Various surgical procedures are available, but recurrence rates of these techniques are disappointingly high. The mucosal flap advancement is considered the gold standard for the treatment of high perianal fistula of cryptoglandular origin by most colorectal surgeons. In the literature a recurrence rate between 0 and 63% is reported for the mucosal flap advancement. Recently Armstrong and colleagues reported on a new biologic anal fistula plug, a bioabsorbable xenograft made of lyophilized porcine intestinal submucosa. Their prospective series of 15 patients with high perianal fistula treated with the anal fistula plug showed promising results.

The anal fistula plug trial is designed to compare the anal fistula plug with the mucosal flap advancement in the treatment of high perianal fistula in terms of success rate, continence, postoperative pain, and quality of life.

**Methods/design:**

The PLUG trial is a randomized controlled multicenter trial. Sixty patients with high perianal fistulas of cryptoglandular origin will be randomized to either the fistula plug or the mucosal advancement flap. Study parameters will be anorectal fistula closure-rate, continence, post-operative pain, and quality of life. Patients will be followed-up at two weeks, four weeks, and 16 weeks. At the final follow-up closure rate is determined by clinical examination by a surgeon blinded for the intervention.

**Discussion:**

Before broadly implementing the anal fistula plug results of randomized trials using the plug should be awaited. This randomized controlled trial comparing the anal fistula plug and the mucosal advancement flap should provide evidence regarding the effectiveness of the anal fistula plug in the treatment of high perianal fistulas.

**Trial registration:**

**ISRCTN: **97376902

## Background

A perianal fistula is a common condition. It has an incidence of 5.6 per 100.000 in women and 12.3 per 100.000 in men [1]. The disease occurs predominantly in the third and fourth decade of life [2]. It is believed that infection of the intersphincteric glands is the initiating event in fistula in ano, in a process known as the 'cryptoglandular hypothesis' [3].

Parks *et al. *[4] developed a classification system in which fistula are divided into intersphincteric fistula, transsphincteric fistula, suprasphincteric fistula and extrasphincteric fistula. However the type of treatment depends not on the location of the fistula tract but of the level of the internal opening in the anal canal.

Low transsphincteric fistulas comprising less than 1/3 of the external sphincter complex are easy to treat by fistulotomy with a high success rate. High transsphincteric fistulas remain a surgical challenge. Surgical procedures include advancement flaps, loose-seton placement, and the installation of fibrin glue. All of these techniques have disappointing success rates. In the literature a recurrence rate between 0 and 63% is reported for the mucosal flap advancement [5-7]. Recently, Van der Hagen *et al. *[6] published the result of 41 patients with high transsphincteric, suprasphincteric and extrasphincteric fistula treated with a mucosal flap advancement. The success rate was a mere 37% (with a median follow-up of 72 months).

The fibrin glue is an alternative to the mucosal advancement flap, however long-term closure rates are low [8-14]. The percentages being as low as 16 percent. The liquid consistency of fibrin glue is possibly not ideal for the purpose of closing anorectal fistulas, because the glue is easily extruded from the fistula tract by increased pressure [15].

Armstrong and colleagues reported a new biologic anal fistula plug [16]. The plug is a FDA and CE approved bioabsorbable xenograft, made of lyophilized porcine intestinal submucosa by Cook Surgical, Inc., Bloomington, IN. The material has inherent resistance to infection, produces no foreign body or giant cell reaction, and becomes repopulated with host cell tissue during a period of three months. The material was fashioned into a conical plug and secured into the primary opening of the fistula tract. Armstrong achieved promising results in a prospective series of 15 patients treated with the anal fistula plug. They compared the results with ten patients using fibrin glue. Patients with high anorectal fistulas (high transsphincteric or deeper) were included. Excluded were patients with Crohn's disease or superficial fistulas (low transsphincteric or more superficial). At a median follow-up of 13.8 ± 3.1 weeks they achieved a significant better fistula closure rate of 87% compared to the fibrin glue group (P < 0.05).

These results call for a prospective randomised controlled trial. Since mucosal flap advancement is the preferred treatment for high cryptoglandular perianal fistula, the anal fistula plug will be compared with mucosal flap advancement in a randomised setting.

## Methods/design

### Study objectives

The objective of this study is to compare, in a prospective randomized way, the anal fistula plug with the mucosal advancement flap in the treatment of high transphincteric perianal fistula in terms of fistula closure rate, continence, morbidity, postoperative pain, and quality of life.

### Study design

The PLUG trial is a prospective double blinded randomized multicenter trial. Patients with high perianal fistulas of cryptoglandular origin will be randomized to either the fistula plug or the mucosal advancement flap. Randomization will be performed during surgery after finding the internal opening. The computer randomization will be done centrally in the Academic Medical Centre in Amsterdam, the Netherlands. Stratification is performed for the randomizing centers.

Patients will be blinded for the type of intervention i.e. anal fistula plug or mucosal advancement flap. Patients are followed-up at two weeks, four weeks, and 16 weeks. At the final follow-up closure rate is determined by clinical examination. Follow-up is done by a colorectal surgeon, who is blinded for the type of intervention. The fistula will be rated closed if the external and the internal opening are closed and no discharge is experienced. Otherwise it is considered as a persistent fistula.

### Study population

The study population consists of patients with high perianal fistulas.

Inclusion criteria are; age above 18 years, high anorectal fistula of cryptoglandular origin (transsphincteric, upper 2/3 of the sphinctercomplex which is confined by the puborectal sling and the end of the anal canal), and informed consent.

Exclusion criteria are; no internal opening found during surgery, HIV-positive patients, Crohn's disease, malignant cause, tuberculosis, hydradenitis suppurativa, and pilonidal sinus disease.

### Primary and secondary endpoints

The primary endpoints of the PLUG trial are fistula closure rate and continence. Continence will be evaluated pre- and postoperatively using the COREFO, the Wexner and the Vaizey score. The COREFO questionnaire has 27 questions to asses colorectal functional outcome [17]. The Vaizey scale consists of three items about the type (gas, fluid, solid) and frequency of incontinence (all scored from zero to four) and four additional items that address alteration in lifestyle (zero to four), the need to wear a pad or plug (zero or two), the use of constipating medication (zero or two), and the lack of ability to defer defecation for 15 minutes (zero or four). The total score on the Vaizey scale ranges from 0 (complete continence) to 24 (complete incontinence) [18].

Secondary endpoints are morbidity, postoperative pain, and quality of life. Postoperatively patients will be asked to grade their pain on a visual analogue scale (VAS: 0, no pain; 10, worst imaginable pain) on different moments during the follow-up. Quality of life will be evaluated using the SF-36 questionnaire. The SF-36 measures eight health attributes: physical functioning, social functioning, role limitations due to physical problems, role limitations due to emotional problems, mental health, pain, vitality and general health perception. The higher the score, the better the health rating with 100 points as the maximum for each concept. In addition the EQ-5D questionnaire is used.

### Participating centers

Six Dutch hospitals, including one academic and five non-academic hospitals, will enrol patients.

### Ethics

The study is conducted in accordance with the principles of the Declaration of Helsinki and 'good clinical practice' guidelines. The protocol has been approved by the Medical Ethics Committee of the Academic Medical Centre in Amsterdam and the local Ethical Committees of the participating centers. Prior the randomization informed consent will be obtained from all patients.

### Study outline

Patients presenting in the outpatients department with high perianal fistulas of cryptoglandular origin will be asked for informed consent when the patient fulfils in- and exclusion criteria.

Positioning of the anal fistula plug will be done according to the instructions of Cook SIS technology. The plug is fabricated from Surgisis (Cook Surgical, Inc., Bloomington, IN), a bioabsorbable xenograft, made of lyophilized porcine intestinal submucosa. The material has inherent resistance to infection, produces no foreign body or giant cell reaction, and becomes repopulated with host cell tissue during a period of three months. All procedures will be performed under general or locoregional anaesthesia. Prophylactic broad-spectrum antibiotics will be administered before surgery. During surgery the internal fistula tract opening will be identified, followed by cleaning and debriding the fistula tract with hydrogen peroxide. A suture will be attached to the tail of the plug. A probe is inserted into the external opening exiting through the internal opening and the suture attached to the tail of the plug is grasped. Then the plug is pulled into the fistula tract, tail first. The suture is drawn into the tract until the plug securely blocks the internal opening and fits snugly within the tract. Any remaining portion of the plug that is not implanted in the tract is trimmed and discarded. The internal end of the plug is sutured in place with at least two sutures. The internal sutures should close the anal canal opening. In contrast with former instructions, no external fixation suture is placed. The external opening is left open to allow for drainage of the tract. In case of a wide fistula tract, a second fistula plug can be put in place.

The rectal advancement flap was done according to the following technique. The internal opening was excised followed by mobilization of the mucosa, submucosa, and a small amount of muscular fibers from the internal sphincter complex. A rectal flap with a 2 to 3 cm broad base was mobilized. The rectal flap was mobilized sufficiently to cover the internal opening with overlap. Hemostasis was performed to prevent a hematoma under the flap. The fistula tract was curetted. The internal opening was not closed before advancing the flap over the internal opening. Finally the flap was sutured in the distal anal canal.

### Statistical analysis

#### Intention to treat

The analysis will be performed in accordance with the intention to treat principle.

#### Sample size calculation

A success percentage of 87% was reported by Armstrong and colleagues for the anal fistula plug [16]. For the mucosal advancement flap a success percentage of 37% was reported recently by Hagen and colleagues in a series of 41 patients with a follow-up of 72 months [6].

To detect an increase in success percentage from 40% to 80%, using a significance level of 0.05, at least 46 patients have to be randomized to achieve a power of 80%. In total, 60 patients will be randomized.

### Data collection and monitoring

Data are collected via datasheets on paper, which are sent to the Academic Medical Centre by mail. Postoperatively questionnaires on pain are filled in by patients. Sixteen weeks after surgery questionnaires are sent to the patients to assess continence and quality of life.

There will be regular contact between the study coordinators and the participating centers. One research fellow will monitor the included data of every patient.

## Discussion

The main objective in the treatment of perianal fistula is the healing of the fistula by closing the internal opening while preserving the anal continence. Submucosal, intersphincteric, and low transsphincteric fistulas, in the lower one-third of the external sphincter complex are easy to treat by simple fistulotomy, with a favourable success rate and relatively little impact on faecal continence [19,20]. The surgical treatment of high perianal fistulas of cryptoglandular origin in relation to morbidity remains a difficult problem. The anal fistula plug appears to be a promising alternative to the current treatment options for high perianal fistulas [20-24]. The anal fistula plug is fabricated from porcine collagen which stimulates tissue remodelling leading to full closure the fistulous tract. Furthermore, the advantage of the plug is that it can be used repeatedly, without risk of damaging the anal sphincter. In a recent study describing the results of the surgical treatment of perianal fistulas of cryptoglandular origin soiling was reported following surgery in 40% of the patients [25]. In these series 109 patients were treated by fistulotomy for low perianal fistulas. A rectal advancement flap was performed in 70 patients for high transsphincteric fistulas. Another advantage is that installing the plug is minimally invasive with possibly less postoperative pain. During mucosal flap advancement, the fistula tract is excised externally to the anal sphincter in order to ensure an optimal drainage.

Before broadly implementing the anal fistula plug results of randomized trials using the plug should be awaited. This randomized controlled trial comparing the anal fistula plug and the mucosal advancement flap should provide evidence regarding the effectiveness of the anal fistula plug in the treatment of high perianal fistulas.

## Abbreviations

VAS: Visual Analogue Scale; SF-36: Short Form-36; EQ-5D: EuroQol; COREFO: Colorectal functional outcome.

## Competing interests

The authors declare that they have no competing interests.

## Authors' contributions

PJvK drafted the manuscript and designed the trial. JFMS, WAB, and PMMB designed the trial and co-authored the writing of the manuscript. All other authors are local investigators at the participating centers and have participated in the design of the trial. All authors co-authored the manuscript and approved the final version.

## Pre-publication history

The pre-publication history for this paper can be accessed here:


